# Semantic Richness Effects in Syntactic Classification: The Role of Feedback

**DOI:** 10.3389/fpsyg.2016.01394

**Published:** 2016-09-15

**Authors:** Melvin J. Yap, Penny M. Pexman

**Affiliations:** ^1^Department of Psychology, National University of SingaporeSingapore, Singapore; ^2^Department of Psychology, University of CalgaryCalgary, AB, Canada

**Keywords:** stimulus quality, semantic richness, visual word recognition, syntactic classification, semantic feedback, RT distributional analyses

## Abstract

Words with richer semantic representations are recognized faster across a range of lexical processing tasks. The most influential account of this finding is based on the idea that semantic richness effects are mediated by feedback from semantic-level to lower-level representations. In an earlier lexical decision study, Yap et al. (2015) tested this claim by examining the joint effects of stimulus quality and four semantic richness dimensions (imageability, number of features, semantic neighborhood density, semantic diversity). The results of that study showed that joint effects of stimulus quality and richness were generally additive, consistent with the idea that semantic feedback does not typically reach the earliest levels of representation in lexical decision. The present study extends this earlier work by investigating the joint effects of stimulus quality and the same four semantic richness dimensions on syntactic classification performance (is this a noun or verb?), which places relatively more emphasis on semantic processing. Additive effects of stimulus quality and richness were found for two of the four targeted dimensions (concreteness, number of features) while semantic neighborhood density and semantic diversity did not seem to influence syntactic classification response times. These findings provide further support against the view that semantic information reaches early letter-level processes.

## Introduction

In order to understand the mechanisms and processes that support reading, researchers have examined the effect of a myriad of word properties on lexical processing performance (see Yap and Balota, 2015, for a review). However, although the ultimate goal of reading is comprehension, the visual word recognition literature has traditionally been dominated by studies that consider the influence of orthographic (e.g., bigram frequency, word length, frequency of occurrence, orthographic neighborhood density), phonological (e.g., regularity, consistency), and morphological (e.g., morphological family size, derivational, and inflectional entropy) characteristics on tasks such as lexical decision (i.e., discriminating between a word and nonwords such as FLIRP) and speeded pronunciation (i.e., reading letter strings aloud). In addition to these variables, there is increasing evidence that *semantic richness* (i.e., the extent to which words are associated with relatively more semantic information) is also an important predictor of word recognition performance (see Pexman, 2012, for a review).

Across standard lexical processing paradigms, including lexical decision, speeded pronunciation, perceptual identification (i.e., identifying visually degraded stimuli), and semantic decision (e.g., classifying words as animate or inanimate), it is now well-established that semantically rich words are generally recognized more quickly and accurately (Pexman et al., 2008; Yap et al., 2012). We should point out here that semantic richness should not be considered a unitary construct, but is instead most appropriately reflected by a number of dimensions which map onto distinct theoretical perspectives.

These dimensions include, but are not limited to, the *number of semantic features* participants associate with a word's referent (e.g., COW's features include *has four legs, eats grass, produces milk*; McRae et al., 2005), its *semantic neighborhood density* (Shaoul and Westbury, 2010), its *number of senses* (Miller, 1990; Hoffman et al., 2013), the *number of distinct first associates* elicited by the word in free association (Nelson et al., 1998), *imageability* or *concreteness*, the extent to which the word evokes mental imagery (Cortese and Fugett, 2004; Brysbaert et al., 2014), *body-object interaction*, the extent to which a human body can interact with the word's referent (Siakaluk et al., 2008), *sensory experience ratings*, the extent to which a word evokes a sensory or perceptual experience (Juhasz and Yap, 2013), modality-specific *perceptual strength*, the extent to which a word's referent is experienced through the five senses (Lynott and Connell, 2009; Connell and Lynott, 2014), and *emotional valence* (i.e., whether a word is positive, negative, or neutral; Yap and Seow, 2014). While investigators typically focus on one semantic richness variable at a time, there have been attempts to characterize the relative predictive power of different dimensions. For instance, Yap et al. (2012) compared the influence of number of features, number of senses, semantic neighborhood density, imageability, and body-object interaction across multiple word recognition tasks. While every variable produced significant effects in at least one task, only the effects of imageability and number of features were reliable (or borderline reliable) across all tasks, indicating that imaginal and featural aspects may be weighted relatively more heavily in a word's semantic representation.

## Richness effects through semantic feedback

Collectively, the foregoing findings converge on the idea that the word recognition system has access to a word's meaning *before* a word is fully identified (Balota, 1990). The theoretical framework most commonly used to explain this finding is an embellished version of the interactive activation and competition (IAC) model of letter perception (McClelland and Rumelhart, 1981). The IAC model contains processing nodes that are organized at three levels (features, letters, words) and is both *interactive* (i.e., activation can flow bidirectionally between levels) and *cascaded* (i.e., as soon as processing at a level begins, it sends activation to the next level). Cascaded processing contrasts with *thresholded* processing, in which a later process begins only after an earlier process is complete. By augmenting the standard IAC model with meaning-level representations, Balota (1990; see also Balota et al., 1991) suggested that semantic influences in word recognition can be accommodated by feedback from semantic-level to lexical-level (i.e., word-level) representations. Specifically, semantically richer words (e.g., words with many semantic features) generate more semantic-level activity, thereby producing stronger feedback to lexical-level units. If one further assumes that lexical decision and speeded pronunciation responses are driven by lexical-level orthographic and phonological activity, respectively, the semantic feedback received by lexical-level units will consequently speed up lexical decision and pronunciation times (Hino and Lupker, 1996; Pexman et al., 2002).

Although studies have explored feedback from semantic- to lexical-level representations (Pexman et al., 2002) and from phonological to orthographic representations (Pexman et al., 2001), the extent to which semantic richness effects are mediated by word-to-letter feedback is less well-understood. The top-down influence of word- on letter-level representations is an integral assumption of McClelland and Rumelhart's (1981) IAC model, and remains a fundamental aspect of the field's most influential word recognition models, including the dual-route cascaded (DRC) model (Coltheart et al., 2001), the multiple read-out model (Grainger and Jacobs, 1996), the bimodal interactive activation framework (Grainger et al., 2005), and the CDP+ and CDP++ models (Perry et al., 2007, 2010). On a related note, the interaction between semantic priming and target degradation (i.e., stronger semantic priming when targets are visually degraded, e.g., Balota et al., 2008) has also been explained using semantic feedback to letter-level representations by way of lexical-level representations (McNamara, 2005).

To test the assumption that meaning-level information reaches the letter level, Yap et al. (2015), using the lexical decision task, investigated the joint effects of stimulus quality (clear vs. degraded) with four richness dimensions, imageability, number of features, semantic neighborhood density, and ambiguity, which map onto distinct and influential theoretical perspectives (see Pexman, 2012, for more discussion). Presenting words in a degraded manner slows down early feature- and letter-level processing (Blais et al., 2011), and interactive effects of stimulus quality and a factor (e.g., semantic richness) indicate that the factor exerts an influence on an early processing locus (see, Sternberg, 1998, for more discussion of additive factors logic). If semantic richness effects indeed reflect partially activated letter-level representations, the most straightforward prediction is that the deleterious impact of visual degradation should be smaller for words which are semantically richer. Interestingly, Yap et al. (2015) did not observe this pattern. Instead, they found robust *additive* effects of stimulus quality and richness (i.e., two main effects and no interaction) for the targeted dimensions. In other words, degradation effects were equivalent in magnitude for words that were high and low in semantic richness. In the light of these findings, Yap et al. (2015) suggested that semantic feedback does not appear to reach earlier levels of representation in lexical decision. Additionally, accommodating the additive effects of stimulus quality and richness seems to require a more complex theoretical account wherein activation is thresholded at the letter level but cascaded from the lexical level onwards (Besner and Roberts, 2003; Reynolds and Besner, 2004).

Yap et al. (2015) proposed that their findings are also consistent with a flexible lexical processor (Balota and Yap, 2006) which adaptively modulates the processing dynamics of early word recognition processes (i.e., whether letter-level processing is cascaded or thresholded) in response to task contexts and demands. In lexical decision, the ultimate goal of the participant is to discriminate between familiar/meaningful real words and unfamiliar/meaningless nonwords, making familiarity an important dimension for word-nonword discrimination (Balota and Chumbley, 1984). Stimulus degradation may undermine such familiarity-based information (Yap and Balota, 2007), and thresholding the letter output helps to recover the familiarity signal by perceptually normalizing degraded stimuli.

## The present study

The results from Yap et al. (2015) show quite clearly that the effects of stimulus quality and richness are additive in lexical decision. However, as discussed above, it is possible that this pattern is idiosyncratic to lexical decision, because of the task's emphasis on familiarity-based information. The first goal of the present study was to explore if the additive effects of stimulus quality and richness generalize to a syntactic classification task (is this word a noun or verb?), a task which demands more extensive consideration of the word's meaning (see Sidhu et al., 2014, for more discussion of task demands). The richness dimensions of interest are similar to those studied in Yap et al. (2015) and include concreteness, number of features, semantic neighborhood density, and ambiguity. Experiment 1 examines the effects of concreteness and number of features, while Experiment 2 examines the effects of semantic neighborhood density and ambiguity.

Unlike lexical decision, which is primarily driven by the familiarity of the orthographic code (Balota and Chumbley, 1984), syntactic classification reflects the ease with which semantic coding can be completed (Hino et al., 2006). If letter-level thresholding is indeed a flexible and adaptive consequence of lexical decision's heavy reliance on familiarity-based information, then such thresholding (and its attendant additive effects) may be absent in the syntactic classification task, which places less emphasis on orthographic familiarity. Instead, one might predict an interaction between stimulus quality and semantic richness in syntactic classification, with smaller degradation effects for richer targets. We should also point out that uninflected verb stimuli will be used in the present study, that is, “verbness” cannot be simply assessed by a superficial check for diagnostic morphemes or suffixes. Instead, participants need to judge if a word's meaning denotes actions or entities, which likely requires more semantic processing than standard lexical decision.

More importantly perhaps, there is evidence that the nature of semantic richness effects varies across tasks. For example, there is a theoretically intriguing dissociation in the literature, where ambiguous words are associated with a processing *advantage* in lexical decision (Borowsky and Masson, 1996) but a processing *disadvantage* in semantic decision (Piercey and Joordens, 2000). Multiple meanings produce greater feedback from semantic- to lexical-level representations, which is helpful in lexical decision. However, in a task which relies more heavily on the semantic code, multiple meanings can slow responses down due to one-to-many mappings from orthography to semantics (Borowsky and Masson, 1996), greater competition between different meanings (Grainger et al., 2001), or competition between the activated meanings and the required response (Pexman et al., 2004). Thus far, task dissociations have been studied at the level of main effects. For example, Hino et al. (2002) examined how the main effect of semantic ambiguity varied across three lexical processing tasks, lexical decision, speeded naming, and semantic categorization. Our second goal is to determine if similar dissociations are observable for the *joint* effects of stimulus quality and the different semantic richness dimensions.

In order to characterize our effects in a more fine-grained manner, we will examine our data both at the level of mean response times (RTs) and at the level of RT distributional characteristics (Balota and Yap, 2011). Specifically, empirical RT distributions will be fitted to the ex-Gaussian function (Heathcote et al., 1991), a convolution of a normal and exponential distribution. Such an analysis yields three parameter estimates: μ and σ (mean and standard deviation of the normal distribution) and τ (mean of the exponential distribution). Along with quantile plots, which provide a graphical representation of distributional effects, ex-Gaussian analysis helps determine the extent to which semantic richness effects in syntactic classification are reflected by distributional shifting (μ) and/or an increase in the tail of the distribution (τ). More relevantly for the present study, there is evidence that spurious additivity in means can be driven by opposing interactive effects in the underlying distribution. For example, Yap et al. (2008) found additive effects of stimulus quality and word frequency at the level of the mean that were due to the combination of an overadditive interaction in μ (reflecting modal RTs) and an underadditive interaction in τ (reflecting slowest RTs). The distributional analyses will therefore help us to rule out such trade-offs in our data. More broadly, the present analyses help extend our earlier work by providing complementary insights into the influence of semantic richness in a task which places greater weight on semantic processing.

## Experiment 1

### Method

#### Participants

Thirty-two undergraduates from the University of Calgary participated for partial course credit. Participants reported in a pre-screening survey that their first language was English; they also had normal or corrected-to-normal vision.

#### Design

Two 2 × 2 designs were incorporated within the same experiment, with non-overlapping items used to examine the effects of each variable. Specifically, we examined stimulus quality (clear or degraded) × concreteness (high or low) and stimulus quality × number of features (high or low). All variables were manipulated within-participants and the dependent variables were RTs and accuracy rates.

#### Stimuli

A total of 240 nouns were selected, with 120 words (60 high and 60 low) each for concreteness and number of features. To determine whether a word is a noun, we examined its part of speech in the English Lexicon Project (Balota et al., 2007; http://elexicon.wustl.edu) and selected words that were coded only as NN (i.e., noun); we avoided words (e.g., CAN) which can be used both as a noun and a verb. Concreteness ratings were based on the norms collected by Brysbaert et al. (2014). Number of features values were taken from McRae et al. (2005). Word sets in each of the experimental conditions were matched on number of letters, number of syllables, number of morphemes, orthographic neighborhood size, and log-transformed subtitle-based contextual diversity (Brysbaert and New, 2009; see Table 1 for descriptive statistics). Additionally, words in the two levels of concreteness were matched on semantic diversity and semantic neighborhood size^1^, while words in the two levels of number of features were matched on concreteness, semantic diversity, and semantic neighborhood size. Using the Match program (Van Casteren and Davis, 2007), an additional 240 verbs (120 for each semantic richness dimension) were selected from the English Lexicon Project to serve as distracters; these were matched as closely as possible to the nouns on number of letters, number of syllables, orthographic neighborhood size, and frequency. While there was no significant difference (*p*s > 0.2) between nouns and verbs on number of letters, orthographic neighborhood size, and number of syllables, nouns (*M* = 2.21) were slightly higher in frequency than verbs (*M* = 2.08). We should also note that verbs and nouns were not explicitly matched on semantic properties (e.g., concreteness); this will be further addressed in the General Discussion.

**Table 1:** **Descriptive statistics for the noun and verb stimuli used in Experiment 1**.

	**High concreteness (*N* = 60)**		**Low concreteness (*N* = 60)**	
	**Mean**	***SD***	**Mean**	***SD***
**CONCRETENESS**				
Concreteness	4.92	0.06	2.89	0.65
Number of letters	5.90	1.53	5.92	1.29
Number of syllables	1.75	0.57	1.80	0.44
Number of morphemes	1.17	0.53	1.10	0.30
Frequency	2.26	0.44	2.24	0.54
Orthographic neighborhood size	2.25	3.67	1.97	3.76
Semantic diversity	1.50	0.17	1.54	0.24
ARC	0.55	0.09	0.57	0.09
	**High no. of features (*N* = 60)**		**Low no. of features (*N* = 60)**	
	**Mean**	***SD***	**Mean**	***SD***
**NUMBER OF FEATURES**				
Number of features	14.58	2.12	9.08	1.82
Number of letters	5.87	1.79	6.38	2.02
Number of syllables	1.85	0.76	2.07	0.82
Frequency	2.22	0.40	2.14	0.39
Orthographic neighborhood size	3.05	4.46	2.12	3.76
Semantic diversity	1.42	0.23	1.42	0.24
ARC	0.52	0.10	0.51	0.09
Concreteness	4.85	0.21	4.81	0.18
	**Mean**	***SD***		
**VERBS (*****N*** = **240)**				
Number of letters	6.21	1.66		
Number of syllables	1.86	0.70		
Frequency	2.08	0.58		
Orthographic neighborhood size	5.14	8.08		

*Frequency, log^10^ transformed subtitle contextual diversity (Brysbaert and New, 2009); Orthographic neighborhood size, number of words that can be formed by substituting a single letter in the target word (Coltheart et al., 1977); ARC, semantic neighborhood density (Shaoul and Westbury, 2010)*.

#### Procedure

Computers running E-prime software (Schneider et al., 2001) were used to present stimuli and collect data. Participants were tested individually in sound-attenuated cubicles, and positioned about 60 cm from the monitor. They were instructed to decide if the word presented formed a noun or verb by making the appropriate button press response (slash key for nouns and Z key for verbs). Participants were encouraged to respond quickly but not at the expense of accuracy. The 20 practice trials were followed by six experimental blocks of 80 trials each, with breaks between blocks. Additionally, the order in which stimuli were presented was randomized anew for each participant. Stimuli were presented in uppercase 18-point Courier New, and each trial comprised the following events: (a) a fixation point (+) at the center of the monitor for 400 ms, (b) a blank screen for 200 ms, and (c) the target. The target remained on the screen for 4000 ms or until a response was made. If a response was incorrect, a 170 ms tone was presented simultaneously with the word “Incorrect” displayed slightly below the fixation point for 450 ms. The same degradation procedure used in Yap et al. (2015) was adopted, i.e., half the targets were degraded by rapidly alternating letter strings with a randomly generated mask of the same length. For example, the mask @$#&% was presented for 14 ms, followed by a five-letter target word for 28 ms, and the two rapidly alternated until a response was detected. Mask patterns were consistent within a trial, and were generated from random permutations of the following symbols: &@?!$^*^%#?. Across participants, targets were counterbalanced across degraded and clear conditions.

### Results and discussion

Trials with response errors (7.9% of trials) were first excluded from the analyses. Noun responses faster than 200 ms or slower than 3000 ms (0.8% of responses) were then eliminated before a mean and standard deviation was computed for each participant as a function of stimulus quality. RTs beyond 2.5 SDs from each participant's mean were excluded, removing a further 2.0% of the responses. Estimates for ex-Gaussian parameters (μ, σ, τ) were obtained using the quantile maximum likelihood estimation (QMLE) procedure in the QMPE program (Version 2.18; Cousineau et al., 2004). All fits converged successfully within 250 iterations. The mean RTs, accuracy rates, and ex-Gaussian parameters are presented in Table 2. Using the lme4 package (Bates et al., 2015), RT effects were analyzed using linear mixed effects (LME) models while accuracy effects were analyzed using generalized linear mixed (GLM) models; *p*-values for fixed effects were obtained using the lmerTest package (Kuznetsova et al., 2016). The main and interactive effects of stimulus quality and semantic richness were treated as fixed effects. Effect coding was used, whereby clear and degraded words were, respectively, coded as −0.5 and 0.5, and words high and low on semantic richness were, respectively, coded as −0.5 and 0.5. Random intercepts for participants and targets, along with by-participant and by-target random slopes for stimulus quality, were included in each model. To the extent models could converge, the by-participant random slope for the relevant semantic richness variable was also included.

**Table 2:** **Mean RTs and accuracy rates as a function of concreteness/number of features and stimulus quality**.

	**RT**	**Accuracy**	**μ**	**σ**	**τ**
**HIGH CONCRETENESS**					
Clear	796 (21)	0.96 (0.008)	554 (11)	54 (7)	247 (15)
Degraded	925 (24)	0.95 (0.007)	633 (14)	70 (9)	297 (20)
Stimulus quality effect	129	0.01	79	16	50
**LOW CONCRETENESS**					
Clear	945 (32)	0.86 (0.013)	668 (21)	113 (16)	284 (27)
Degraded	1095 (33)	0.86 (0.017)	739 (23)	119 (16)	370 (27)
Stimulus quality effect	150	0.00	71	6	86
**HIGH NUMBER OF FEATURES**					
Clear	772 (19)	0.98 (0.005)	556 (11)	57 (7)	217 (11)
Degraded	901 (24)	0.97 (0.007)	618 (15)	52 (8)	287 (16)
Stimulus quality effect	129	0.01	62	−5	70
**LOW NUMBER OF FEATURES**					
Clear	804 (20)	0.97 (0.007)	563 (11)	52 (6)	241 (17)
Degraded	950 (27)	0.97 (0.006)	635 (12)	48 (7)	323 (21)
Stimulus quality effect	146	0.00	72	−4	82

*Standard errors are in parentheses*.

Table 3 presents the results for the joint effects of stimulus quality with concreteness. For RTs, the main effects of concreteness (*p* < 0.001), and stimulus quality (*p* < 0.001) were both significant. RTs were faster for high-concreteness (*M* = 864 ms) than for low-concreteness (*M* = 1029 ms) nouns, and faster for clear (*M* = 877 ms) than for degraded (*M* = 1015 ms) nouns. The stimulus quality × concreteness interaction was not significant. Comparing the additive model (two main effects) to the interactive model (two main effects and an interaction) did not reveal a significant difference in their likelihood, $\chi_{\left(1\right)}^{2}$ = 0.327, *ns*. For accuracy, only the main effect of concreteness (*p* < 0.001) was significant; accuracy was higher for high-concreteness (*M* = 0.95) than for low-concreteness (*M* = 0.86) nouns.

**Table 3:** **LME (top panel: RT) and GLM (bottom panel: Accuracy) model estimates for fixed and random effects for the joint effects of stimulus quality with concreteness**.

**Random effects**	**Variance**	***SD***	***r***
**ITEMS**			
Intercept	6563.70	81.02	
Stimulus quality	339.40	18.42	
**PARTICIPANTS**			
Intercept	19272.40	138.83	
Stimulus quality	2409.30	49.08	
Concreteness	4436.00	66.60	0.52
**Fixed effects**	**Coefficient**	**Standard error**	***p*****-value**
Intercept	946.25	26.21	<0.001
Concreteness	165.10	21.87	<0.001
Stimulus quality	138.00	14.04	<0.001
Stimulus quality × concreteness	12.58	22.08	NS
**Random effects**	**Variance**	***SD***	***r***
**ITEMS**			
Intercept	1.09	1.04	
Stimulus quality	0.00	0.00	
**PARTICIPANTS**			
Intercept	0.24	0.49	
Stimulus quality	0.02	0.15	
Concreteness	0.49	0.70	1.00
**Fixed effects**	**Coefficient**	**Standard error**	***p*****-value**
Intercept	2.98	0.16	<0.001
Concreteness	−1.41	0.28	<0.001
Stimulus quality	−0.15	0.14	NS
Stimulus quality × concreteness	0.38	0.29	NS

We now turn to the ex-Gaussian parameters. For μ, the main effect of concreteness was significant, *F*_*p*__(1, 31)_ = 90.91, *p* < 0.001, *MSE* = 4251.25, η_*p*_^2^ = 0.75; μ was greater for low-concreteness (*M* = 703 ms) than for high-concreteness (*M* = 593 ms) nouns. The main effect of stimulus quality was significant, *F*_*p*__(1, 31)_ = 21.97, *p* < 0.001, *MSE* = 8147.09, η_*p*_^2^ = 0.42; μ was greater for degraded (*M* = 686 ms) than for clear (*M* = 611 ms) nouns. The stimulus quality × concreteness interaction was not significant, *F* < 1. For σ, only the effect of concreteness was significant, *F*_*p*__(1, 31)_ = 25.75, *p* < 0.001, *MSE* = 3669.62, η_*p*_^2^ = 0.45; σ was greater for low-concreteness (*M* = 116 ms) than for high-concreteness (*M* = 62 ms) nouns. Finally, for τ, the main effects of concreteness, *F*_*p*__(1, 31)_ = 11.37, *p* = 0.002, *MSE* = 8480.01, η_*p*_^2^ = 0.27, and stimulus quality, *F*_*p*__(1, 31)_ = 18.43, *p* < 0.001, *MSE* = 8038.08, η_*p*_^2^ = 0.37, were significant; τ was greater for low-concreteness (*M* = 327 ms) than for high-concreteness (*M* = 272 ms) nouns, and greater for degraded (*M* = 333 ms) than for clear (*M* = 265 ms) nouns. The stimulus quality × concreteness interaction was not significant, *F* < 1.

**Figure 1:**
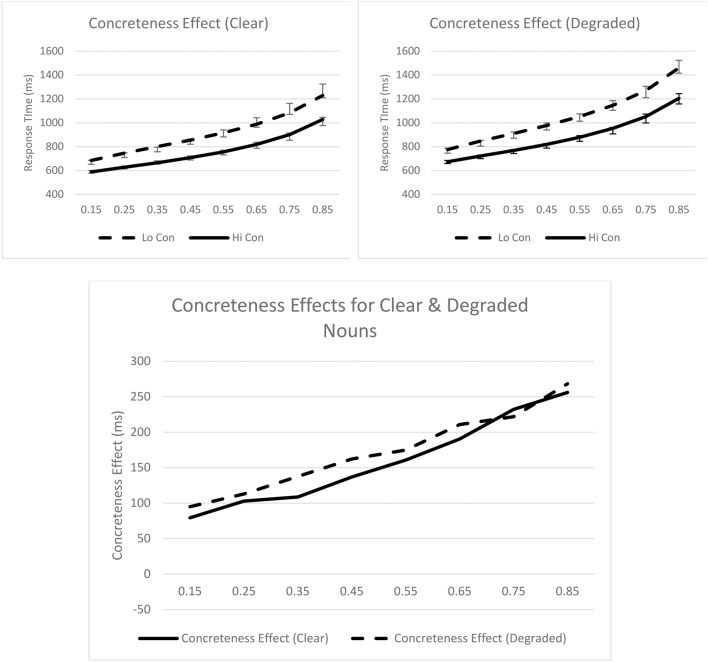
**Syntactic classification performance as a function of concreteness and quantiles for clear (top left panel) and degraded (top right panel) words**. Empirical quantiles are represented by error bars, whereas fitted ex-Gaussian quantiles are represented by lines. The bottom panel shows concreteness effects as a function of stimulus quality. Con, concreteness.

To illustrate these effects graphically, the mean quantiles (0.15, 0.25, 0.35, 0.45, 0.55, 0.65, 0.75, 0.85) for the different experimental conditions are plotted on Figure 1. In the top two panels of the figure, the empirical quantiles are represented by data points and error bars, while the theoretical quantiles for the best-fitting ex-Gaussian distribution are represented by lines. The bottom panel of the figure represents concreteness effects as a function of stimulus quality. In general, the empirical data were well-captured by the ex-Gaussian parameters; empirical and theoretical quantiles generally did not diverge by more than one standard error.

#### Number of features

Table 4 presents the results for the joint effects of stimulus quality with number of features. For RTs, the main effects of number of features (*p* = 0.019) and stimulus quality (*p* < 0.001) were both significant. RTs were faster for nouns with more features (*M* = 838 ms) than for nouns with fewer features (*M* = 879 ms); they were also faster for clear (*M* = 789 ms) than for degraded (*M* = 928 ms) nouns. The stimulus quality × concreteness interaction was not significant. Comparing the additive model to the interactive model did not reveal a significant difference in their likelihood, $\chi_{\left(1\right)}^{2}$ = 0.758, *ns*. For accuracy, none of the effects were statistically significant.

**Table 4:** **LME (top panel: RT) and GLM (bottom panel: Accuracy) model estimates for fixed and random effects for the joint effects of stimulus quality with number of features**.

**Random effects**	**Variance**	***SD***	***r***
**ITEMS**			
Intercept	6390.39	79.94	
Stimulus quality	2135.66	46.21	
**PARTICIPANTS**			
Intercept	13098.23	114.45	
Stimulus quality	2539.68	50.40	
Number of features	48.53	6.97	1.00
**Fixed effects**	**Coefficient**	**Standard error**	***p*-value**
Intercept	858.36	22.02	<0.001
Number of features	41.59	17.42	0.019
Stimulus quality	139.33	13.63	<0.001
Stimulus quality × number of features	17.89	20.64	NS
**Random effects**	**Variance**	***SD***	***r***
**ITEMS**			
Intercept	1.22	1.11	
Stimulus quality	9.42 × 10−10	3.07 × 10−5	
**PARTICIPANTS**			
Intercept	0.24	0.49	
Stimulus quality	0.74	0.86	
Number of features	0.11	0.33	0.12
**Fixed effects**	**Coefficient**	**Standard error**	***p*-value**
Intercept	4.29	0.23	<0.001
Number of features	−0.24	0.32	NS
Stimulus quality	−0.36	0.26	NS
Stimulus quality × number of features	0.25	0.48	NS

**Figure 2:**
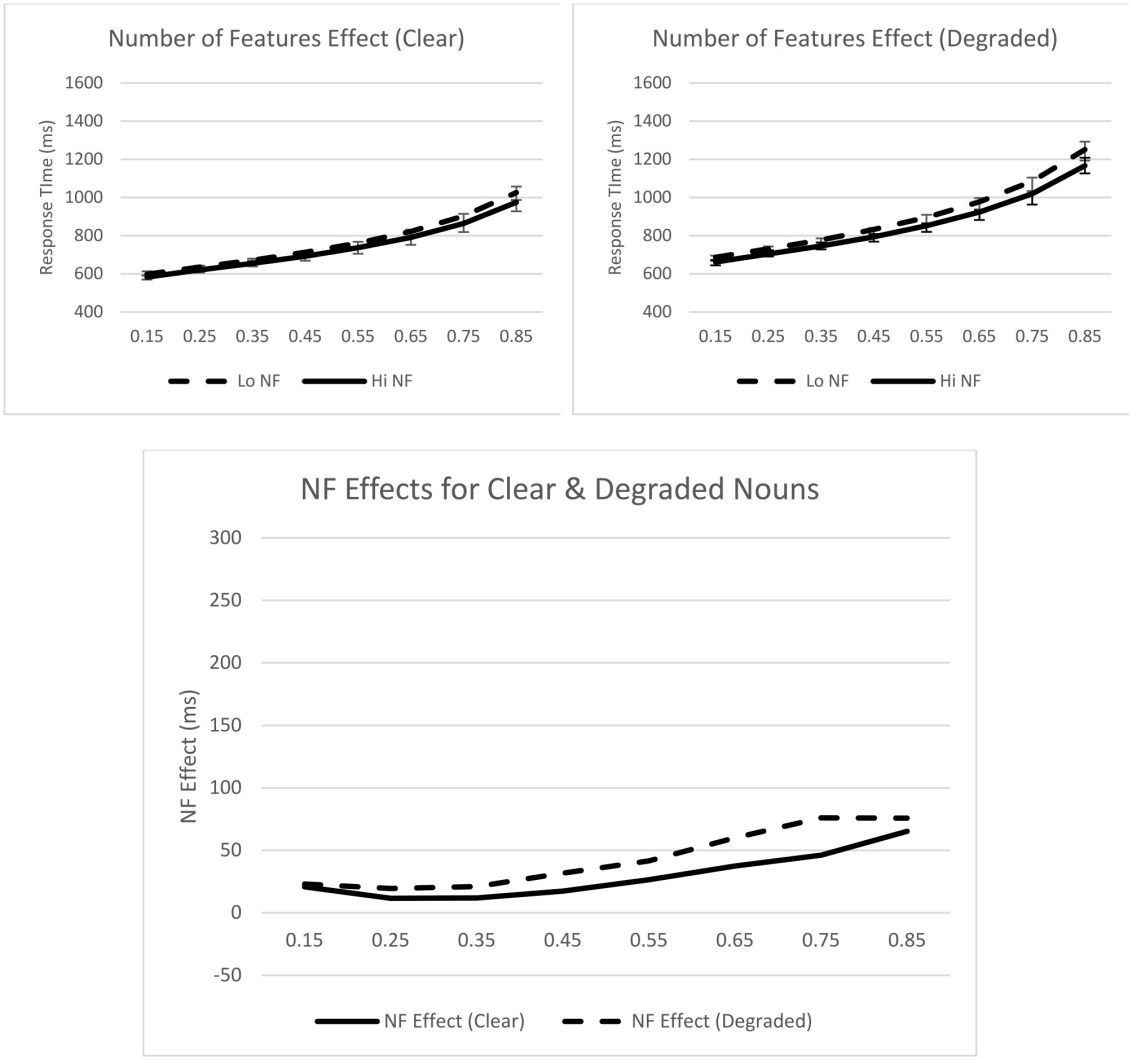
**Syntactic classification performance as a function of number of features and quantiles for clear (top left panel) and degraded (top right panel) words**. Empirical quantiles are represented by error bars, whereas fitted ex-Gaussian quantiles are represented by lines. The bottom panel shows number of feature effects as a function of stimulus quality. NF, number of features.

Turning to the ex-Gaussian parameters, for μ, only the main effect of stimulus quality was significant, *F*_*p*__(1, 31)_ = 73.34, *p* < 0.001, *MSE* = 1952.19, η_*p*_^2^ = 0.70; μ was greater for degraded (*M* = 626 ms) than for clear (*M* = 560 ms) nouns. The stimulus quality × number of features interaction was not significant, *F* < 1. For σ, none of the effects were significant, *F*s < 1. Finally, for τ, the main effects of number of features, *F*_*p*__(1, 31)_ = 5.43, *p* = 0.026, *MSE* = 5316.32, η_*p*_^2^ = 0.15, and stimulus quality, *F*_*p*__(1, 31)_ = 29.83, *p* < 0.001, *MSE* = 6156.83, η_*p*_^2^ = 0.49, were significant; τ was greater for nouns with fewer features (*M* = 282 ms) than for nouns with more features (*M* = 252 ms), and greater for degraded (*M* = 305 ms) than for clear (*M* = 229 ms) nouns. The stimulus quality × concreteness interaction was not significant, *F* < 1. These effects are graphically represented in Figure 2.

#### Summary

In Experiment 1, reliable additive effects of stimulus quality and semantic richness were observed in RTs. That is, responses were faster for clear nouns and for semantically rich nouns, but richness effects were not statistically different in magnitude for clear and degraded nouns. The supplementary distributional analyses indicated that the stimulus quality × semantic richness interaction was not significant for any ex-Gaussian parameter, confirming that the additive patterns in mean RTs were not qualified by trade-offs between distributional parameters. There are a couple of other noteworthy observations. In Yap et al.'s (2015) lexical decision study, richness effects were generally mediated by a combination of distributional shifting (μ) and an increase in the tail of the distribution (τ). In the present study, while this pattern was indeed observed for concreteness effects, the influence of number of features was predominantly reflected in τ. Interestingly and unexpectedly, the main effect of concreteness (*M* = 165 ms) was much larger than the main effect of number of features (*M* = 41 ms); we will comment on this further in the General Discussion.

## Experiment 2

### Method

#### Participants

Thirty-two undergraduates from the University of Calgary participated for partial course credit. Participants reported in a pre-screening survey that their first language was English; they also had normal or corrected-to-normal vision. Participants who had taken part in Experiment 1 were not allowed to participate in Experiment 2.

#### Design

Like E1, two 2 × 2 designs were incorporated within the experiment: Stimulus Quality × Semantic Neighborhood Density (dense or sparse) and Stimulus Quality × Ambiguity (high or low). All variables were manipulated within-participants and the dependent variables were RTs and accuracy rates.

#### Stimuli

A total of 240 nouns were selected, with 120 nouns (60 high and 60 low) each for semantic neighborhood density and ambiguity. Semantic neighborhood density was operationally defined by average radius of co-occurrence (ARC; Shaoul and Westbury, 2010), which refers to the mean of the distance between the target word and all neighbors within a pre-specified threshold; higher ARC values indicate denser neighborhoods. Ambiguity was operationally defined by Hoffman et al.'s (2013) recently developed *semantic diversity* measure, which estimates semantic ambiguity by tracking the variability in the contextual usage of words; words with higher values on semantic diversity are more ambiguous.

Experimental conditions were matched on the same control lexical variables described in Experiment 1 (see Table 5 for descriptive statistics). Additionally, words in the two levels of semantic neighborhood density were matched on concreteness and semantic diversity, while words in the two levels of semantic diversity were matched on concreteness and semantic neighborhood size. As in Experiment 1, the Match program (Van Casteren and Davis, 2007) was used to select an additional 240 distracter verbs that were matched as closely as possible to the nouns on number of letters, number of syllables, orthographic neighborhood size, and frequency. There was no significant difference (*p*s > 0.38) between nouns and verbs on number of letters, orthographic neighborhood size, and number of syllables; however, nouns (*M* = 2.17) were marginally higher in frequency than verbs (*M* = 2.08), *p* = 0.05.

**Table 5:** **Descriptive statistics for the noun stimuli used in Experiment 2**.

**Word stimuli**	**High neighborhood density (*N* = 60)**		**Low neighborhood density (*N* = 60)**	
	**Mean**	***SD***	**Mean**	***SD***
**SEMANTIC NEIGHBORHOOD DENSITY**				
ARC	0.62	0.02	0.42	0.06
Number of letters	5.95	1.38	6.02	1.41
Number of syllables	1.75	0.54	1.73	0.52
Number of morphemes	1.17	0.38	1.23	0.43
Frequency	2.23	0.29	2.19	0.29
Orthographic neighborhood size	2.27	3.75	2.27	3.72
Semantic diversity	1.45	0.17	1.42	0.17
Concreteness	4.39	0.64	4.46	0.60
**Word stimuli**	**High semantic diversity (*N* = 60)**		**Low semantic diversity (*N* = 60)**	
	**Mean**	***SD***	**Mean**	***SD***
**SEMANTIC DIVERSITY**				
Semantic diversity	1.81	0.09	1.06	0.15
Number of letters	5.63	1.34	5.80	1.26
Number of syllables	1.67	0.57	1.77	0.59
Frequency	2.16	0.44	2.09	0.41
Orthographic neighborhood size	2.37	3.40	2.00	3.06
ARC	0.55	0.11	0.54	0.07
Concreteness	4.14	0.79	4.15	0.72
	**Mean**	***SD***		
**VERBS (*N* = 240)**				
Number of letters	5.96	1.38		
Number of syllables	1.73	0.55		
Frequency	2.08	0.56		
Orthographic neighborhood size	2.23	3.54		

*Orthographic neighborhood size, number of words that can be formed by substituting a single letter in the target word (Coltheart et al., 1977); Frequency, log^10^ transformed subtitle contextual diversity (Brysbaert and New, 2009); Concreteness, concreteness ratings (Brysbaert et al., 2014); ARC, average radius of co-occurrence, a measure of semantic neighborhood density (Shaoul and Westbury, 2010)*.

#### Procedure

Same as Experiment 1.

### Results and discussion

As in Experiment 1, trials with response errors (10.6% of trials) were first excluded from the analyses. Noun responses faster than 200 ms or slower than 3000 ms (0.7% of responses) were then eliminated before a mean and standard deviation was computed for each participant as a function of stimulus quality. RTs beyond 2.5 *SD*s from each participant's mean were excluded, removing a further 2.1% of the responses. The mean RTs, accuracy rates, and ex-Gaussian parameters are presented in Table 6.

**Table 6:** **Mean RTs and accuracy rates as a function of semantic neighborhood density/semantic diversity and stimulus quality**.

	**RT**	**Accuracy**	**μ**	**σ**	**τ**
**HIGH NEIGHBORHOOD DENSITY**					
Clear	820 (29)	0.93 (0.011)	583 (22)	63 (10)	242 (21)
Degraded	943 (35)	0.92 (0.011)	636 (14)	51 (7)	315 (27)
Stimulus quality effect	123	0.01	53	−12	73
**LOW NEIGHBORHOOD DENSITY**					
Clear	831 (28)	0.93 (0.013)	586 (14)	56 (9)	249 (19)
Degraded	965 (34)	0.92 (0.011)	641 (14)	62 (7)	331 (26)
Stimulus quality effect	134	0.01	55	6	82
**HIGH SEMANTIC DIVERSITY**					
Clear	826 (26)	0.86 (0.014)	585 (17)	68 (9)	247 (18)
Degraded	981 (39)	0.82 (0.015)	643 (15)	60 (7)	350 (32)
Stimulus quality effect	155	0.04	58	−8	103
**LOW SEMANTIC DIVERSITY**					
Clear	833 (27)	0.94 (0.010)	590 (14)	61 (7)	249 (20)
Degraded	987 (37)	0.92 (0.012)	659 (16)	76 (10)	332 (29)
Stimulus quality effect	154	0.02	69	15	83

*Standard errors are in parentheses*.

#### Semantic neighborhood density

Table 7 presents the results for the joint effects of stimulus quality with semantic neighborhood density. For RTs, only the main effect of stimulus quality (*p* < 0.001) was significant; RTs were faster for clear (*M* = 830 ms) than for degraded (*M* = 957 ms) nouns. Comparing the model with only a main effect of stimulus quality to the additive model did not reveal a significant difference in their likelihood, $\chi_{\left(1\right)}^{2}$ = 1.018, *ns*. For accuracy, none of the effects were statistically significant.

**Table 7:** **LME (top panel: RT) and GLM (bottom panel: Accuracy) model estimates for fixed and random effects for the joint effects of stimulus quality with semantic neighborhood density**.

**Random effects**	**Variance**	***SD***	
**ITEMS**			
Intercept	6300.00	79.37
Stimulus quality	370.40	19.25
**PARTICIPANTS**			
Intercept	27391.90	165.50
Stimulus quality	4726.70	68.75
**Fixed effects**	**Coefficient**	**Standard error**	***p*****-value**
Intercept	893.75	30.58	<0.001
Semantic neighborhood density	17.88	17.76	NS
Stimulus quality	127.02	15.98	<0.001
Stimulus quality × semantic neighborhood density	8.89	20.73	NS
**Random effects**	**Variance**	***SD***	
**ITEMS**			
Intercept	1.26	1.12
Stimulus quality	0.00	0.00
**PARTICIPANTS**			
Intercept	0.50	0.71
Stimulus quality	0.33	0.57
**Fixed effects**	**Coefficient**	**Standard error**	***p*****-value**
Intercept	3.18	0.19	<0.001
Semantic neighborhood density	−0.10	0.25	NS
Stimulus quality	−0.15	0.17	NS
Stimulus quality × semantic neighborhood density	0.04	0.25	NS

**Figure 3:**
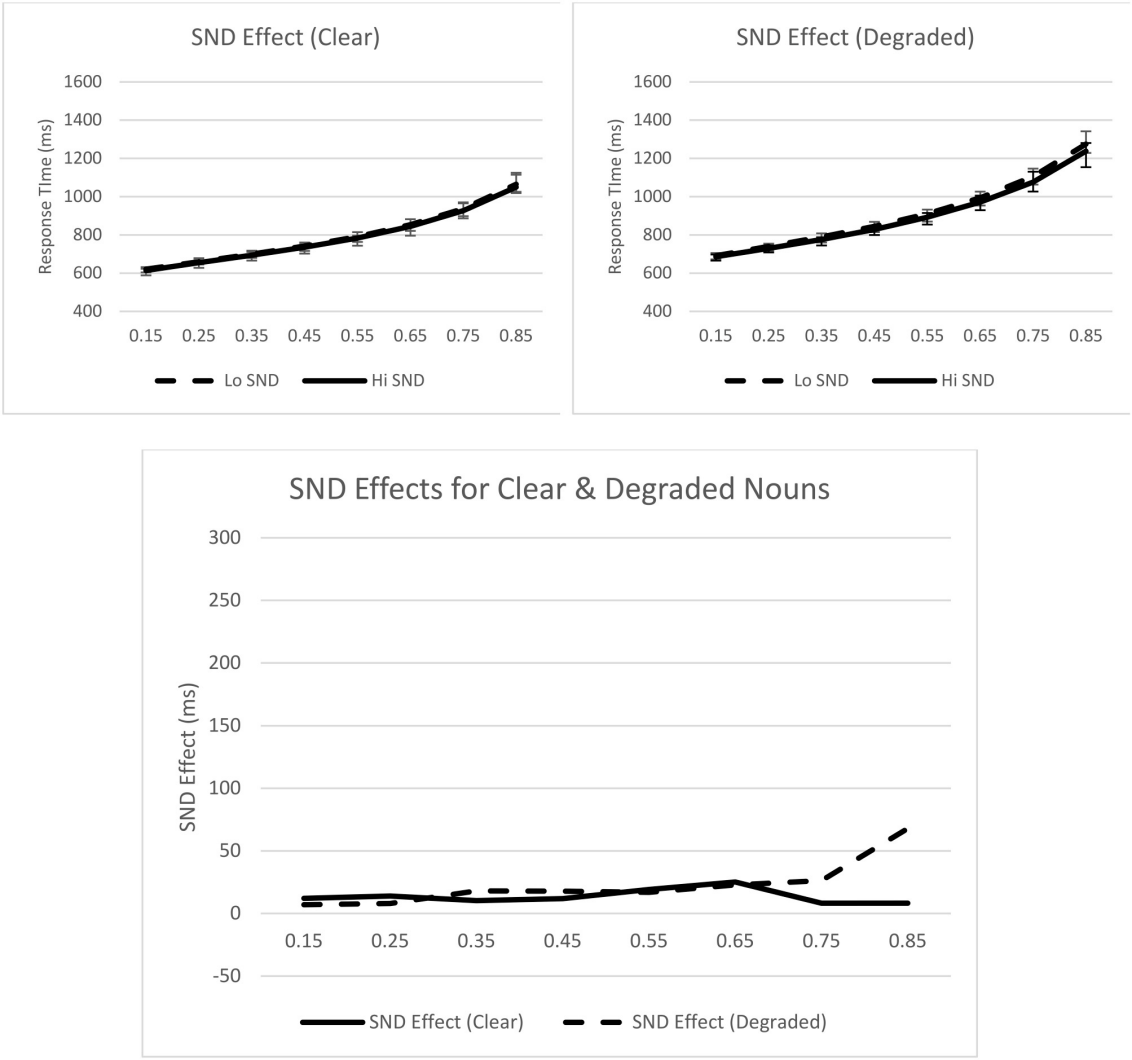
**Syntactic classification performance as a function of semantic neighborhood density and quantiles for clear (top left panel) and degraded (top right panel) words**. Empirical quantiles are represented by error bars, whereas fitted ex-Gaussian quantiles are represented by lines. The bottom panel shows semantic neighborhood density effects as a function of stimulus quality. SND, semantic neighborhood density.

Turning to the ex-Gaussian parameters, for μ, only the main effect of stimulus quality was significant, *F*_*p*__(1, 31)_ = 26.91, *p* < 0.001, *MSE* = 3476.21, η_*p*_^2^ = 0.46; μ was greater for degraded nouns (*M* = 639 ms) than for clear nouns (*M* = 585 ms). For σ, none of the effects were significant. Finally, for τ, only the main effect of stimulus quality was significant, *F*_*p*__(1, 31)_ = 21.57, *p* < 0.001, *MSE* = 8871.92, η_*p*_^2^ = 0.41; τ was greater for degraded nouns (*M* = 323 ms) than for clear nouns (*M* = 245 ms). These effects are graphically represented in Figure 3.

#### Semantic diversity

Table 8 presents the results for the joint effects of stimulus quality with semantic diversity. For RTs, only the main effect of stimulus quality (*p* < 0.001) was significant; RTs were faster for clear (*M* = 836 ms) than for degraded (*M* = 992 ms) nouns. Comparing the model with only a main effect of stimulus quality to the additive model did not reveal a significant difference in their likelihood, $\chi_{\left(1\right)}^{2}$ = 0.024, *ns*. For accuracy, both the main effects of stimulus quality (*p* = 0.003) and semantic diversity (*p* = 0.002) were significant. Accuracy was higher for clear (*M* = 0.90) than for degraded (*M* = 0.87) nouns, and higher for *less* ambiguous (i.e., low semantic diversity) nouns (*M* = 0.93), compared to *more* ambiguous (i.e., high semantic diversity) nouns (*M* = 0.84).

**Table 8:** **LME (top panel: RT) and GLM (bottom panel: Accuracy) model estimates for fixed and random effects for the joint effects of stimulus quality with semantic diversity**.

**Random effects**	**Variance**	***SD***
**ITEMS**			
Intercept	8041.10	89.67
Stimulus quality	401.50	20.04
**PARTICIPANTS**			
Intercept	29332.00	171.27
Stimulus quality	4392.80	66.28
**Fixed effects**	**Coefficient**	**Standard error**	***p*****-value**
Intercept	914.12	31.82	<0.001
Semantic diversity	3.00	19.59	NS
Stimulus quality	156.55	15.89	<0.001
Stimulus quality × semantic diversity	−1.05	21.46	NS
**Random effects**	**Variance**	***SD***
**ITEMS**			
Intercept	1.92	1.39
Stimulus quality	5.63 × 10−14	2.37 × 10−7
**PARTICIPANTS**			
Intercept	0.38	0.62
Stimulus quality	0.00	0.00
**Fixed effects**	**Coefficient**	**Standard error**	***p*****-value**
Intercept	2.87	0.19	<0.001
Semantic diversity	0.89	0.29	0.002
Stimulus quality	−0.34	0.12	0.003
Stimulus quality × semantic diversity	0.18	0.23	NS
			

**Figure 4:**
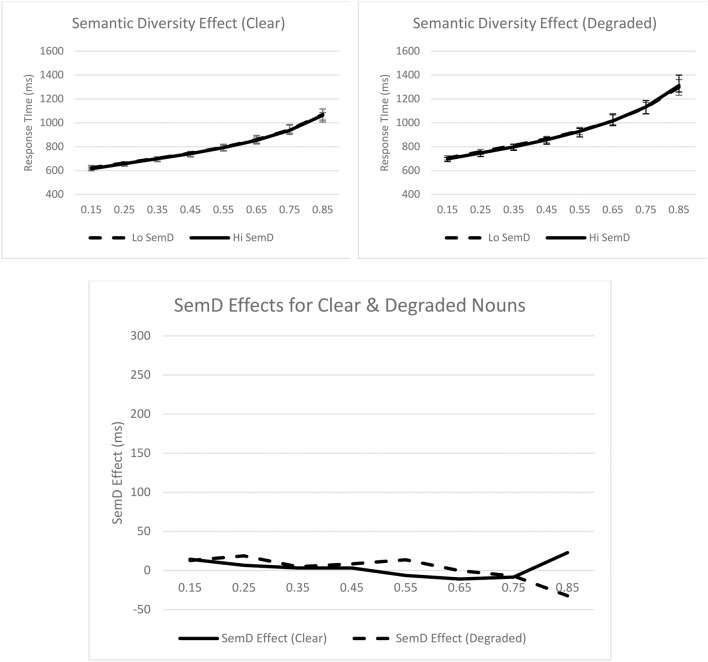
**Syntactic classification performance as a function of semantic diversity and quantiles for clear (top left panel) and degraded (top right panel) words**. Empirical quantiles are represented by error bars, whereas fitted ex-Gaussian quantiles are represented by lines. The bottom panel shows semantic diversity effects as a function of stimulus quality. SemD, semantic diversity.

Turning to the ex-Gaussian parameters, for μ, only the main effect of stimulus quality was significant, *F*_*p*__(1, 31)_ = 43.86, *p* < 0.001, *MSE* = 2885.90, η_*p*_^2^ = 0.59; μ was greater for degraded nouns (*M* = 651 ms) than for clear nouns (*M* = 588 ms). For σ, none of the effects were significant. Finally, for τ, only the main effect of stimulus quality was significant, *F*_*p*__(1, 31)_ = 22.82, *p* < 0.001, *MSE* = 12251.46, η_*p*_^2^ = 0.42; τ was greater for degraded (*M* = 341 ms) than for clear (*M* = 248 ms) nouns. These effects are graphically represented in Figure 4.

#### Summary

Compared to Experiment 1, semantic richness effects in Experiment 2 were far less robust. Specifically, semantic neighborhood density had no effect on RT or accuracy rates, while the influence of semantic diversity was restricted to accuracy rates. Importantly, as in the previous experiment, there was no evidence that these effects were qualified by stimulus quality, either in the mean RTs or in the underlying RT distributional characteristics. To establish the robustness of the null findings in Experiment 2, we conducted supplementary analyses to examine the effects of concreteness, semantic neighborhood density, and semantic diversity, using newly available megastudy data from the Calgary semantic decision project (Pexman et al., 2016). In this megastudy, participants were required to classify words as concrete or abstract. In total, semantic decision RTs and accuracy rates were collected for 5000 concrete and 5000 abstract words from 321 participants. For present purposes, we conducted a simultaneous multiple regression analysis for 2451 concrete words which were associated with an accuracy rate of at least 70%. The predictors included control lexical variables (number of letters, number of syllables, number of morphemes, orthographic neighborhood size, word frequency), along with concreteness, semantic neighborhood density, and semantic diversity^2^. For RTs, the effects of semantic neighborhood density (*p* = 0.13) and semantic diversity (*t* < 1) were not significant. However, there was an effect of concreteness, with faster responses to concrete words (β = −0.51, *p* < 0.001, *sr*^2^ = 0.24). Turning to accuracy rates, the effects of all three variables were significant or approached significance. Concrete words (β = 0.51, *p* < 0.001, *sr*^2^ = 0.24) and words in dense neighborhoods (β = 0.05, *p* = 0.015, *sr*^2^ = 0.002) were responded to more accurately, while ambiguous words (β = −0.04, *p* = 0.052, *sr*^2^ = 0.001) were responded to less accurately. These regression analyses, although based on an independent abstract/concrete semantic decision dataset, are broadly consistent with the key findings in Experiment 2.

#### Combined analyses

We conducted an additional analysis in which RT data from both experiments were combined, in order to statistically compare the magnitude of richness effects for different dimensions. To do this, all words high on richness (e.g., high concreteness, high number of features, high neighborhood density, high diversity) were coded as −0.5, while words low on richness were coded as 0.5. As before, clear and degraded words were, respectively, coded as −0.5 and 0.5. Table 9 presents the results for this combined analysis. The main effects of stimulus quality (*p* < 0.001) and semantic richness (*p* < 0.001) were significant, but there was no interaction. Following this, we created six contrast codes corresponding to the six possible pairwise comparisons between the four dimensions (C1: concreteness vs. number of features; C2: concreteness vs. semantic neighborhood density; C3: concreteness vs. semantic diversity; C4: number of features vs. semantic neighborhood density; C5: number of features vs. semantic diversity; C6: semantic neighborhood density vs. semantic diversity). For each contrast, we tested a model where the joint effects of stimulus quality, richness, and the respective contrast code were examined. Richness interacted significantly with C1 (*p* < 0.001), C2 (*p* < 0.001), and C3 (*p* < 0.001), but not with the other contrast codes. In other words, although the effects of concreteness were significantly larger than the effects of the other three variables, there was no significant difference between the effects of number of features, semantic neighborhood density, and semantic diversity. This suggests that although the effect of number of features was statistically significant in the individual analyses, while the effects of semantic neighborhood density and semantic diversity were not, this distinction did not hold up in the composite analysis. That being said, our study was designed to separately test the joint effects of stimulus quality with different semantic richness dimensions, and most likely lacks the statistical power to adequately compare the magnitude of different semantic richness effects. This question can be explored more systematically in future research based on more powerful designs.

**Table 9:** **LME model estimates for fixed and random effects for the joint effects of stimulus quality with semantic richness (composite analysis)**.

**Random effects**	**Variance**	***SD***	***r***
**ITEMS**			
Intercept	8666.90	93.10	
Stimulus quality	1019.80	31.93	
**PARTICIPANTS**			
Intercept	21522.00	146.70	
Stimulus quality	2932.40	54.15	
Concreteness	567.30	23.82	0.20
**Fixed effects**	**Coefficient**	**Standard error**	***p*****-value**
Intercept	904.68	19.05	<0.001
Semantic richness	49.65	9.97	<0.001
Stimulus quality	140.31	8.65	<0.001
Stimulus quality × semantic richness	10.45	10.71	NS

## General discussion

In the present study, we examined the joint effects of stimulus quality and four semantic richness dimensions (concreteness, number of features, semantic neighborhood density, semantic diversity) in verb/noun syntactic classification. Our primary objective was to ascertain if the additive effects of stimulus quality and semantic richness previously reported in lexical decision (Yap et al., 2015) generalized to a different binary decision task which is not familiarity-based, and which places more emphasis on semantic processing. With respect to this basic question at least, our results are clear-cut. There was no evidence for an interaction between stimulus quality and any of the targeted richness dimensions, either in mean RTs or in the RT distributional characteristics. In other words, the additive effects of stimulus quality and semantic richness cannot be fully attributed to the specific demands of lexical decision. That being said, the study also yielded a number of other findings which are more surprising and less straightforward. For example, semantic diversity (a measure of ambiguity) had no effect on RTs, but ambiguous words were associated with lower accuracy rates. On the other hand, concreteness effects were atypically large compared to the effects of number of features and semantic neighborhood density. These findings will now be discussed at greater length.

### Semantic richness effects: the role of feedback

As mentioned in the Introduction, the semantic feedback account has been a popular perspective for accommodating semantic richness effects. Although not usually articulated, there is an underlying assumption that meaning-level activation also reaches the letter level by way of orthographic and phonological representations. Indeed, this fundamental assumption continues to inform influential computational models of visual word recognition (e.g., DRC, multiple read-out, CDP+) that incorporate the interactive activation model (McClelland and Rumelhart, 1981) as a cornerstone. Complementing the empirical observation of additive effects of stimulus quality and richness in lexical decision (Yap et al., 2015), the additive patterns reported by the present study provide further evidence against the view that feedback from semantics is able to reach earlier levels of representation in visual word recognition.

The notion that letter-level processing is not modulated by semantic information meshes well with some recent findings from the semantic priming domain. Specifically, there is a well-known overadditive interaction between stimulus quality and semantic priming, wherein degradation effects are larger for related (e.g., cat—DOG), compared to unrelated (e.g., hat—DOG), prime-target pairs (Meyer et al., 1975). One explanation for this interaction is that the prime word (e.g., CAT) activates related words (e.g., DOG) through spreading activation, and through feedback, there is prospective pre-activation of the lexical- and letter-level representations of the related words, thus attenuating the deleterious impact of degradation. This account has been undermined by a study by Thomas et al. (2012), who examined the stimulus quality × priming interaction for different types of prime-target pairs. Forward asymmetric pairs (e.g., keg—BEER) have a prime-to-target association but no target-to-prime association, backward asymmetric pairs (e.g., small—SHRINK) have a target-to-prime association but no prime-to-target association, and symmetric prime-target pairs (e.g., cat—DOG) are related in both directions. The key finding was that the stimulus quality × priming interaction was reliable only for pairs with a target-to-prime association (i.e., symmetric and backward asymmetric pairs), suggesting that the interaction was carried by a retrospective strategic process that depended on a relationship from the target to the prime. For our purposes, these results cannot be reconciled with an account based on a prospective semantic feedback mechanism, since that would predict an interaction for pairs with a prime-to-target association (i.e., symmetric and forward asymmetric pairs).

As discussed in the Introduction, additivity in computational models can be achieved by implementing thresholded output from the letter level (Besner and Roberts, 2003; Reynolds and Besner, 2004). However, why would letter-level processing be thresholded in the syntactic classification task? We do not have a definitive answer here, but suggest that the results may reflect a flexible lexical processor that is responsive to task context and demands, and which modulates processing pathways in order to optimize performance on a task (Balota et al., 1999; Balota and Yap, 2006; Tousignant and Pexman, 2012). As mentioned in the Introduction, uninflected verb stimuli were used in the present study. Therefore, in order to produce a correct response on the syntactic classification task, it is necessary to precisely identify a specific lexical representation to determine if its meaning denotes an action or entity.

Letter-level thresholding, which allows degraded stimuli to be normalized to match perceptually clear stimuli (Yap and Balota, 2007), can reduce the likelihood of a degraded letter string activating the meaning of an incorrect candidate. The impact of such an error would be profound and particularly difficult to recover from in syntactic classification. Our account is conceptually inspired by O'Malley and Besner's (2008) proposal that there is letter-level thresholding in the speeded pronunciation task when participants have to name both words and nonwords. By “cleaning up” the stimulus, thresholding minimizes the possibility of *lexical capture*, whereby degraded nonwords may activate a word sufficiently strongly such that readers mistakenly read it as a word instead of the nonword.

### Task-specificity of semantic richness effects

The present study was designed to extend the earlier lexical decision study by Yap et al. (2015) by examining the joint effects of the same variables in syntactic classification. There were some noteworthy differences in the results of the two studies. Specifically, in lexical decision, all four semantic richness dimensions (imageability, number of features, semantic neighborhood density, semantic diversity) produced robust effects. In contrast, in syntactic classification RTs, semantic neighborhood density and semantic diversity effects were not reliable. Interestingly, this is not the first time these between-task dissociations have been reported. In order to tease apart task-general from task-specific processing, Yap et al. (2012) evaluated the influence of five semantic richness dimensions (imageability, body-object interaction, ambiguity, semantic neighborhood density, number of features) on five lexical processing tasks (lexical decision, go/no-go lexical decision, speeded pronunciation, progressive demasking, concrete/abstract semantic decision). Importantly, they also found that effects of ambiguity and semantic neighborhood density were not significant in the semantic decision task; Pexman et al. (2008) also failed to find semantic neighborhood effects in semantic categorization. Indeed, these null finding are corroborated by the item-level regression analyses we conducted on the recently published Calgary semantic decision project data (Pexman et al., 2016), which yielded the same pattern of results. In summary, although semantic richness is multidimensional, it is evident that the effect of some of these dimensions (e.g., number of features, concreteness/imageability) are more stable and generalizable across tasks, compared to others (e.g., semantic neighborhood density, semantic diversity) which show greater task-specificity. This suggests that particular facets of a word's semantic representation may carry more weight in influencing lexicosemantic processing.

A few additional aspects of the foregoing findings are instructive. First, semantic neighborhood density effects seem to be relatively variable in tasks which place relatively more weight on semantic processing; they appear in some studies but not in others. There have been suggestions (e.g., Mirman and Magnuson, 2006) that there could be a trade-off between close neighbors (facilitatory effects) and distant neighbors (inhibitory effects); such opposing effects would produce diminished or null effects.

Second, it is reassuring that the analyses on the Calgary megastudy data revealed no effect of semantic diversity on RTs, but an inhibitory effect on accuracy rates (i.e., ambiguous words are less accurately responded to). Yap et al. (2011) also found that ambiguous words were less accurately classified in concrete/abstract semantic decision. Taken together, these trends mirror the findings of the present study, and further support the idea that the facilitation afforded by ambiguity is specific to lexical decision (e.g., Piercey and Joordens, 2000). As discussed earlier, an ambiguity disadvantage is typically reported in tasks which place an emphasis on semantic processing. The modeling work of Hoffman and Woollams (2015) suggests that the high contextual variability associated with semantically diverse words leads to noisy, underspecified semantic representations, which could impede semantic coding. It is unclear why the inhibition afforded by semantic diversity tends to influence accuracy rates, rather than RTs. This is an issue that merits future research.

Finally, based on the semipartial correlations in the supplementary regression analyses, it is clear that the proportion of variance accounted for by concreteness is far greater than that of the other richness dimensions. This was also reported by Pexman et al. (2016), and is consistent with the unusually large concreteness effect observed in Experiment 1. In the Pexman et al. (2016) megastudy, participants had to discriminate between concrete and abstract words, and the concreteness ratings of the concrete words, compared to the abstract words, were, by definition, much higher. This encourages participants to rely on the concreteness dimension to drive the concrete/abstract binary decision, thereby exaggerating the size of concreteness effects. Such a line of reasoning is analogous to participants relying on familiarity-based information in lexical decision, which inflates the size of frequency effects (Balota and Chumbley, 1984). Although Experiment 1 featured the verb/noun rather than concrete/abstract decision, the concreteness ratings were higher for nouns (*M* = 4.41) than for verbs (*M* = 2.81). As a result, it is likely that the concreteness effect was inflated by an emphasis on concreteness information as a discrimination dimension. Moving forward, it is methodologically better if semantic richness properties were more tightly matched for both categories in a semantic decision task. Nonetheless, the important point here is that concreteness effects, large as they were, were not moderated by stimulus quality in the present study. Even in an experimental setting which places such a premium on a particular semantic richness dimension, there is no evidence that this semantic information reaches early letter-level processes.

## Limitations and concluding remarks

We acknowledge that the present results may partly reflect the specific task demands of the syntactic classification task adopted. The decision to use the broad categories of verb and noun was to maximize the number of items that can be presented under the same task demands (see Pexman et al., 2016). However, there is evidence that the decision selected for a semantic task can moderate observed effects (Tousignant and Pexman, 2012; see Pexman et al., 2016, for more discussion). That being said, all other things being equal, researchers (e.g., Jared and Seidenberg, 1991) have recommended using broader, rather than narrower, categories. We are also encouraged by the degree of convergence between the results of Pexman et al. (2016), which used concrete/abstract decision, and the present study, which used verb/noun decision.

Certain methodological aspects of the present work could also be further tightened in future research. While the two levels of semantic richness for each dimension were well-matched on lexical and semantic characteristics, the automated procedure (Van Casteren and Davis, 2007) we used matched nouns and verbs on lexical, but not semantic, variables. Nouns and verbs were not significantly different on number of letters, number of syllables, and orthographic neighborhood size, but nouns, compared to verbs, were slightly higher on frequency. Furthermore, as already discussed, concreteness ratings were higher for nouns than verbs in Experiment 1, which is likely to increase the reliance on concreteness for noun/verb discrimination. However, while verb/noun differences may modulate the emphasis on particular word dimensions for driving binary decisions, this does not qualify the joint effects of stimulus quality with richness, since the counterbalancing procedure ensures that the *same* items were rotated through clear and degraded conditions, and they thus serve as their own control.

Additionally, while the present study focused on four particular semantic richness dimensions (concreteness, number of features, semantic neighborhood density, semantic diversity) in order to facilitate comparisons to our previous lexical decision study (Yap et al., 2015), other richness dimensions of a more embodied nature (e.g., body-object interaction, sensory experience ratings, perceptual strength, emotional valence) remain unstudied in this paradigm and should be the object of future investigations.

Along with the study by Yap et al. (2015), the present work reinforces the claim that one central aspect of the interactive activation framework, i.e., the interactive activation between letter- and lexical-level representations, does not appear to be compatible with how semantic richness effects unfold in visual word recognition. In both lexical decision and syntactic classification, we have observed additive effects of stimulus quality and richness, indicating that the additive pattern cannot be simply explained by lexical decision's emphasis on familiarity-based information. It is possible that the present results reflect a flexible lexical processor that can strategically engage thresholded early processing to optimize task performance. Specifically, we have suggested that thresholding reduces the likelihood that degraded words incorrectly activate the semantics of some other word, but this is speculative and needs to be empirically verified in future investigations.

In sum, the present findings help to further constrain our understanding of the interplay between semantic processing and semantic feedback mechanisms. Our results are consistent with others in the semantic richness literature, in showing that there are multiple dimensions of semantic richness and that these can have different effects both within and between tasks. At a broader level, this study adds to a growing literature showing that lexical semantics is multidimensional, variable, dynamic, and context-sensitive (Pexman et al., 2013).

## Author contributions

MY and PP jointly conceptualized the study, and MY designed the experiments. The data were collected and analyzed by PP and MY, respectively. MY wrote the initial draft of the manuscript, and PP edited and commented on it.

### Conflict of interest statement

The authors declare that the research was conducted in the absence of any commercial or financial relationships that could be construed as a potential conflict of interest. The reviewer RD and handling Editor declared their shared affiliation, and the handling Editor states that the process nevertheless met the standards of a fair and objective review.

## Footnotes

^1^Due to the fact that number of features is available for only a relatively small set of words (*N* = 541), it was not possible to match high- and low-concreteness words on number of features.

^2^We did not include number of features in the regression analysis because this measure is available for a very small set of words, and its inclusion would have greatly reduced the size of the dataset.
